# The RAD51-FFPE Test; Calibration of a Functional Homologous Recombination Deficiency Test on Diagnostic Endometrial and Ovarian Tumor Blocks

**DOI:** 10.3390/cancers13122994

**Published:** 2021-06-15

**Authors:** Lise M. van Wijk, Claire J. H. Kramer, Sylvia Vermeulen, Natalja T. ter Haar, Marthe M. de Jonge, Judith R. Kroep, Cor D. de Kroon, Katja N. Gaarenstroom, Harry Vrieling, Tjalling Bosse, Maaike P. G. Vreeswijk

**Affiliations:** 1 Department of Human Genetics, Leiden University Medical Center, 2300 RC Leiden, The Netherlands; L.M.van_Wijk@lumc.nl (L.M.v.W.); S.Vermeulen@lumc.nl (S.V.); H.Vrieling@lumc.nl (H.V.); 2 Department of Pathology, Leiden University Medical Center, 2300 RC Leiden, The Netherlands; C.J.H.Kramer@lumc.nl (C.J.H.K.); N.T.ter_Haar@lumc.nl (N.T.t.H.); M.M.de_Jonge@lumc.nl (M.M.d.J.); T.Bosse@lumc.nl (T.B.); 3 Department of Medical Oncology, Leiden University Medical Center, 2300 RC Leiden, The Netherlands; J.R.Kroep@lumc.nl; 4 Department of Gynecology, Leiden University Medical Center, 2300 RC Leiden, The Netherlands; C.D.de_Kroon@lumc.nl (C.D.d.K.); K.N.Gaarenstroom@lumc.nl (K.N.G.)

**Keywords:** endometrial carcinoma, ovarian carcinoma, homologous recombination deficiency, RECAP test, RAD51-FFPE test, RAD51, BRCA1, BRCA2

## Abstract

**Simple Summary:**

Rapid and reliable identification of patients with homologous recombination deficient (HRD) tumors is important for treatment choice as these tumors tend to respond well to platinum-based chemotherapy and PARP inhibitors (PARPi). In this study, a RAD51-based functional HRD test that can be performed on routine diagnostic formalin-fixed paraffin-embedded (FFPE) tissues (RAD51-FFPE test), was further improved and optimal test parameters were determined. The RAD51-FFPE test was able to determine tumor HR status with high sensitivity and specificity, making it an attractive test to be applied as routine diagnostic tool in the near future.

**Abstract:**

PARP inhibitor (PARPi) sensitivity is related to tumor-specific defects in homologous recombination (HR). Therefore, there is great clinical interest in tests that can rapidly and reliably identify HR deficiency (HRD). Functional HRD tests determine the actual HR status by using the (dis)ability to accumulate RAD51 protein at sites of DNA damage as read-out. In this study, we further improved and calibrated a previously described RAD51-based functional HRD test on 74 diagnostic formalin-fixed paraffin-embedded (FFPE) specimens (RAD51-FFPE test) from endometrial cancer (EC *n* = 25) and epithelial ovarian cancer (OC *n* = 49) patients. We established optimal parameters with regard to RAD51 foci cut-off (≥2) and HRD threshold (15%) using matched endometrial and ovarian carcinoma specimens for which HR status had been established using a RAD51-based test that required *ex vivo* irradiation of fresh tissue (RECAP test). The RAD51-FFPE test detected *BRCA* deficient tumors with 90% sensitivity and RECAP-HRD tumors with 87% sensitivity, indicating that it is an attractive alternative to DNA-based tests with the potential to be applied in routine diagnostic pathology.

## 1. Introduction

In 2020, the European Society for Medical Oncology (ESMO) published their recommendations regarding applications of predictive biomarkers for homologous recombination deficiency (HRD) detection and subsequent predictive capacity for PARP inhibitors (PARPi) benefit in epithelial ovarian cancer [1]. HRD tests are increasingly important with the recent implementation of PARPi in the first line therapy of patients with high-grade epithelial ovarian cancer [2,3,4,5], besides the use in second line [6,7,8]. Especially in the first line setting, when the platinum free interval of the tumor is not yet known, a rapid and precise HRD test is required. Three approaches for HRD testing were described, i.e., (i) detection of pathogenic genetic alterations in homologous recombination (HR)-related genes or gene silencing by promotor hypermethylation, (ii) genome-based tests: the observation of distinct patterns of somatic mutations due to a defect in HR, including genomic scars and mutational signatures [9,10,11,12,13,14,15], and (iii) determination of real-time HR status by performing functional HRD assays [1]. With respect to the first approach, i.e., detection of pathogenic genetic alterations/gene silencing by promotor hypermethylation in HR-related genes, Konstantinopolous *et al*. reported that an HRD phenotype in terms of (epi)genetic alterations in *BRCA1, BRCA2, PALB2* and additional HR-related genes was observed in approximately 50% of high grade serous epithelial ovarian cancers (HGSOC) [16]. For the second approach, i.e., genome-based tests, multiple studies have indulged in the utilization of genomic scars as a biomarker for HRD [15,17,18,19,20]. Genomic scars include high genomic loss of heterozygosity (LOH), telomeric allelic imbalances (TAI) and large-scale state transitions (LST) [10,13,14,21,22]. Classifiers that are based on genomic scars include the MyChoice^®^ HRD test and HRDetect [15,23]. Both tests are able to detect HRD beyond *BRCA1/2* pathogenic variants; however, the predictive capacity of genomic scar-based HRD tests for therapy response after treatment with PARPi is suboptimal, since the greatest benefit is observed among *BRCA* deficient HRD cases. In addition, PARPi sensitivity could be observed among HR-Proficient (HRP) patients [6,23,24]. This apparent discrepancy may at least partially result from the fact that genome-based approaches capture the genomic history of the tumor instead of the actual HR status of tumors. The latter is especially important in the context of reversion mutations, as has been described for *BRCA1/2* or loss of function variants of the *53BP1* gene that may subsequently lead to restoration of HR function [25,26,27,28,29,30,31].

In the past few years, various *ex vivo* functional tests that determine the actual HR status of tumors have been developed [31,32,33,34,35,36,37,38,39]. The functional REcombination CAPacity (RECAP) test determines the capacity of proliferating tumor cells to accumulate RAD51 protein at ionizing-radiation-induced DNA double strand breaks (DSBs) by co-immunofluorescence staining (co-IF) for RAD51 and geminin (GMN), a marker for the G2/S phase, in which HR can take place [31,40,41]. The RECAP test allowed identification of HRD tumor specimens beyond those carrying *BRCA1/2* pathogenic variants in breast cancer (BC), endometrial cancer (EC) and ovarian cancer (OC) [31,40,41]. Logistic limitations of the RECAP test are, however, the requirement of fresh tumor specimens and the *ex vivo* induction of DNA damage, which complicates implementation of the RECAP test as an HRD test in the clinic.

Interestingly, Cruz *et al.*, recently developed a functional RAD51-based test that can be performed on routine diagnostic formalin-fixed paraffin-embedded (FFPE) BC specimens based on the hypothesis that genomic instability in tumors leads to sufficient high levels of endogenous DNA damage [42,43]. Quantification of yH2AX foci, a marker for DNA damage, confirmed that indeed most BC tumors contained sufficient endogenous DNA damage levels and RAD51 foci were detectable in diagnostic FFPE tumor specimens [42,43]. The ability to detect RAD51 foci in diagnostic FFPE tumor specimens opens the door to its use as a biomarker for HRD in the clinic and in this study, we take two crucial steps towards this goal. Firstly, we optimized co-IF staining protocols and specified quality assessment criteria to establish optimal test parameters for the RAD51-based test on diagnostic FFPE specimens (RAD51-FFPE test) for EC and OC since aforementioned studies have been performed on BC only [42,43]. Secondly, the threshold for functional HRD was calibrated by performing a side-by-side comparison of RAD51-FFPE and previously published RECAP data of matching EC and OC tumor specimens. This resulted in an improved RAD51-FFPE test capable of identifying tumors with functional HRD with high sensitivity, a prerequisite to study the performance of the RAD51-FFPE test as a predictive test for treatment response in future studies.

## 2. Materials and Methods

### 2.1. Patient Material

For this study, archival diagnostic FFPE tumor tissue blocks that matched the tumor specimens of the previously published RECAP test were collected [31,41]. For the RECAP test, fresh endometrial and ovarian tumor specimens were obtained during surgery from patients at the Leiden University Medical Center (LUMC) between June 2010 and July 2017. Clinicopathological characteristics and RECAP scores from the patient cohort used for this study have been previously described in detail in the context of the RECAP test in de Jonge *et al.* and van Wijk *et al*. [31,41]. All specimens were coded with a unique research code. The local medical ethics committee of the LUMC approved the study protocols on 7 February 2011 and 24 May 2017 (B16.019, P10.226 and G17.041), and specimens were handled according to the “Code for Proper Secondary Use of Human Tissue” in the Netherlands as established by the Dutch Federation of Medical Scientific Societies.

### 2.2. RECAP Test

**Figure 1:**
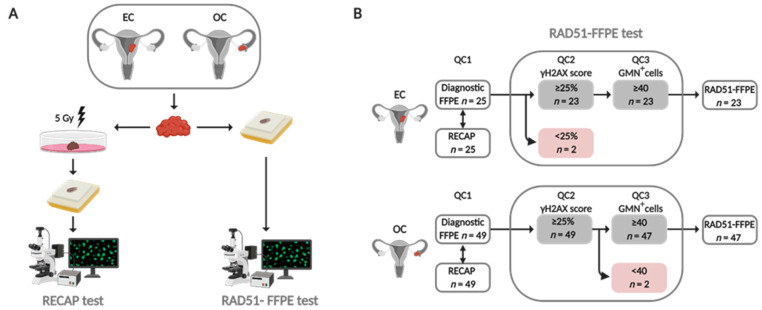
**From RECAP to RAD51-FFPE test. (A) EC and OC tumor specimens obtained during surgery were used for both the RECAP test (left) and RAD51-FFPE test (right).** The RECAP test requires ex vivo irradiation of fresh tumor specimens (5 Gy) and incubation prior to tissue fixation. The RAD51-FFPE test is performed directly on (archival) diagnostic FFPE specimens. (**B**) Diagnostic FFPE specimens matched with RECAP specimens that were previously published [31,41] were subjected to a three-step quality control (QC). First, diagnostic FFPE specimens with >70% tumor tissue were included (QC1). Second, the γH2AX score based on the percentage of γH2AX+/GMN+ (≥ 2 foci) cells was calculated (QC2). If this score was ≥ 25%, specimens were analyzed with a co-IF for the presence of GMN and RAD51. If ≥ 40 GMN+ cells could be counted, the RAD51-FFPE specimen was included for analysis (QC3). Abbreviations: EC = endometrial carcinoma; OC = ovarian carcinoma; RECAP = REcombination CAPacity; FFPE = formalin-fixed paraffin-embedded; GMN = geminin.

In short, fresh tumor specimens, obtained during surgery, were irradiated and incubated for two hours prior to fixation and paraffin embedding (Figure 1A). Irradiated tumor specimens with high tumor percentage and sufficient tumor vitality were included and stained for geminin (GMN; anti-geminin antibody, ProteinTech, Manchester, UK, cat. 10802-1-AP) and RAD51 (anti-RAD51 antibody, GeneTex, Alton Pkwy Irvine, CA, USA, cat. GTX70230) with a co-IF staining. Forty GMN^+^ cells were evaluated for the presence of ≥5 foci/nucleus (RAD51^+^). The percentage of RAD51^+^/GMN^+^ cells was represented as the RECAP score. Tumor specimens were considered HR-Deficient (HRD) with a RECAP score of ≤20%, HR-Intermediate (HRI) with a RECAP score of 21–50% and HR-Proficient (HRP) with a RECAP score of >50%. For the current study, we dichotomized HR-classes (HRP and HRD), for which we considered HRI cases as HRP.

### 2.3. γH2AX/GMN Co-Immunohistochemistry Staining (co-IHC)

Tissue sections (4 µm) were deparaffinized in xylene; endogenous peroxidase was blocked with 0.3% H_2_O_2_, rehydrated in ethanol, heated with target antigen retrieval (10 mM Tris/1 mM EDTA buffer, pH 9.0) in a conventional microwave for 12 min and blocked with wash buffer (DAKO, Agilent, Santa Clara, CA, USA, cat. S3006) with 1% BSA (Bovine Serum Albumin, MilliporeSigma, St. Louis, MO, USA, cat. A7030-100G) for 15 min. The primary γH2AX antibody (mouse, monoclonal, MilliporeSigma, St. Louis, MO, USA, cat. 05-636, clone JBW301) and the primary GMN antibody (rabbit, polyclonal, Proteintech, Manchester, UK, cat. 10802-1-AP) were diluted 1:30.000 and 1:5000 respectively in antibody diluent (DAKO REAL, ready-to-use diluent, Agilent, Santa Clara, CA, USA, cat. S2022) and incubated at room temperature (RT) overnight (o/n). Tissue sections were washed (three times for five minutes) in TBS/Tween and incubated with secondary antibody (BrightVision poly-HRP-anti-mouse, Immunologic, VWR, Amsterdam, The Netherlands, cat. VWRKDPVM110HRP) for 30 min at RT. After washing in TBS/Tween, slides were incubated with liquid chromogen DAB+ (DAKO, Agilent, Santa Clara, CA, USA, K3468) for ten minutes at RT. Tissue sections were washed in MilliQ for five minutes and washed in TBS/Tween. Next, slides were incubated with Donkey anti-Rabbit-AP 1:200 (Abcam, Cambridge, UK, cat. ab7084) 1 h at RT, washed in TBS/Tween and incubated with chromogen Fast Red (Abcam, Cambridge, UK, cat. ab64254). Finally, slides were washed with MilliQ for five minutes, airdried and covered with Surgipath Micromount (Leica Biosystems, Buffalo Grove, IL, USA, cat. 3801731). 

### 2.4. Co-Immunofluorescence (co-IF) Staining for RAD51 and Geminin

An optimized RAD51/GMN co-IF staining protocol was used for diagnostic FFPE specimens, by adapting the protocol of Cruz *et al.* [43]. FFPE tissue sections of 4 µm were incubated in a stove at 63 °C o/n prior to co-IF staining. Tissue sections were deparaffinized in xylene, rehydrated with decreasing concentrations of ethanol (100–90–70%) and washed in MilliQ. Slides were incubated with Antigen Retrieval buffer (DAKO, pH 9.0, Agilent, Santa Clara, CA, USA, cat. S2375) and heated at 97 °C for 12 min using a TissueWave™ 2 Microwave Processor (ThermoFisher Scientific, Waltham, MA, USA). Slides were cooled down for 30 min, washed twice in MilliQ and permeabilized in DAKO wash buffer (DAKO, Agilent, Santa Clara, CA, USA, cat. S3006) for five minutes. Subsequently, the slides were blocked with blocking buffer (DAKO wash buffer with 1% BSA) for ten minutes and incubated with primary antibodies for RAD51 (rabbit, monoclonal, Abcam, Cambridge, UK, cat. ab133534) and GMN (mouse, monoclonal, NovoCastra, Leica Biosystems, Buffalo Grove, IL, USA, cat. NCL-L) (1:1000 and 1:60, respectively) for 60 min at RT. Afterwards, the slides were washed three times with wash buffer for five minutes and incubated with blocking buffer for ten minutes. Slides were incubated with secondary antibodies in blocking buffer, i.e., Goat-anti Mouse IgG Alexa Fluor 488 (Invitrogen, ThermoFisher Scientific, Waltham, MA, USA, cat. A-11001) and Goat-anti Rabbit IgG Alexa Fluor 555 (Invitrogen, ThermoFisher Scientific, Waltham, MA, USA, cat. A-21428) (both diluted 1:500) for 30 min at RT. Slides were washed in wash buffer for five minutes and washed twice in MilliQ for five minutes. After dehydration of the slides with increasing concentrations of ethanol (70–90–100%), slides were mounted with ProLong Gold antifade mountant with DAPI (ThermoFisher Scientific, Waltham, MA, USA, cat. P36935). All slides were stored at −20 °C.

### 2.5. Quality Control

A three-step quality control (QC) was performed on diagnostic FFPE blocks containing tissue (Figure 1B).

First, representative diagnostic FFPE blocks containing >70% vital tumor tissue were selected by a gynecopathologist (T.B.) (QC1).

Second, the presence of endogenous DNA damage in tumor cells of selected FFPE specimens was confirmed by evaluation of a γH2AX/GMN co-IHC (QC2) (Figure S1). At least 40 GMN^+^ cells, randomly selected in 3–5 vital tumor tissue areas, were manually counted by two independent observers on a Zeiss Axio Imager.M2 light microscope, 63× oil objective. The number of γH2AX foci were counted per selected GMN^+^ cell (0, 1, 2, 3, 4 or ≥5 γH2AX foci). The γH2AX score was determined by calculation of the average percentage of γH2AX^+^/GMN^+^ cells (cut-off ≥2 γH2AX foci) of two observers. Diagnostic FFPE tumor specimens with a γH2AX score <25% were excluded for analysis due to the lack of sufficient endogenous DNA damage for HR to be visualized.

Third, the presence of sufficient GMN^+^ cells was confirmed based on a GMN/RAD51 co-IF (QC3). Since different GMN primary antibodies were used for the co-IHC (QC2) and the co-IF, we included QC3 to be certain that sufficient GMN^+^ cells could be counted for the calculation of the RAD51-FFPE score (Section 2.6). At least 40 GMN^+^ cells, randomly selected in 3–5 vital tumor tissue areas, were considered sufficient. Tumor specimens with <40 GMN^+^ cells in the co-IF were excluded for analysis.

### 2.6. RAD51-FFPE Score Calculation

Diagnostic FFPE tumor tissue sections were stained for DAPI, GMN and RAD51 in a co-IF staining and scored manually with a Leica DM6B microscope, 63×/1.40–0.6 oil objective with an EL6000 light source. DAPI was used to get an overall impression of the whole tumor section, assess cell morphology and locate three to five areas of the tumor enriched with vital tumor cells. Within vital tumor areas, GMN^+^ cells were identified and ≥40 GMN^+^ cells were selected at random. A cell was considered GMN^+^ when the nucleus was completely stained with a granular pattern. The number of RAD51 foci within a GMN^+^ cell was determined (0, 1, 2, 3, 4 or ≥5 foci) and cells were categorized accordingly. For each RAD51 foci cut-off, a RAD51-FFPE score was calculated as the percentage of RAD51^+^/GMN^+^ cells by each observer. Final RAD51-FFPE scores were calculated as the average RAD51-FFPE score of two independent observers. When the difference of RAD51-FFPE scores in a tumor specimen was >30% between two observers, a third independent observer was consulted to generate a final score (Figure S2). In the case of a clear sub-optimal staining, i.e., strong autofluorescence, aspecific RAD51 staining or strong pan-nuclear RAD51 staining, final scores were calculated as the average of the two closest RAD51-FFPE scores. Sub-optimal staining was observed in nine OC specimens. When tumor heterogeneity was observed, i.e., discrete areas with either RAD51^+^/GMN^+^ cells or RAD51^-^/GMN^+^ cells, scores of three independent observers were averaged. This was the case for one EC specimen. In total, for ten out of 70 diagnostic FFPE specimens (OC *n* = 9, EC *n* = 1), a third observer was consulted.

### 2.7. Genetic and Epigenetic Analyses

Genetic analyses (*BRCA1/2* next-generation sequencing (NGS); HR gene panel) and epigenetic analyses (*BRCA1* promoter hypermethylation by MS-MLPA) were previously performed by de Jonge *et al*. and van Wijk *et al.* and data were obtained from these studies [31,41].

### 2.8. Statistical Analysis

Figures were created with Graphpad Prism 8.0 (GraphPad Software, San Diego, CA, USA), Adobe Illustrator CC 2020 (Adobe Inc, San Jose, CA, USA) and BioRender software (Toronto, ON, Canada). Statistical analysis was performed with Graphpad Prism 8.0 and SigmaStat 3.5 (Systat Software Inc, San Jose, CA, USA). Student’s *t*-tests were performed to test differences between two groups containing normally distributed numerical data and Mann-Whitney Rank Sum tests when numerical data was not normally distributed. Categorical data of two groups were tested with Chi-square test or Fisher’s exact test. Fisher’s exact test was chosen when at least one of the expected values was less than one and when over 20% of the expected values were less than five. To test if numerical data was correlated between two groups, Pearson’s correlation coefficient was calculated. The Cohen kappa coefficient (k) was used to measure interobserver and intertest agreement. A *p*-value of < 0.05 was considered significant.

## 3. Results

### 3.1. Diagnostic FFPE Specimen Inclusion and Quality Control (QC)

**Figure 2:**
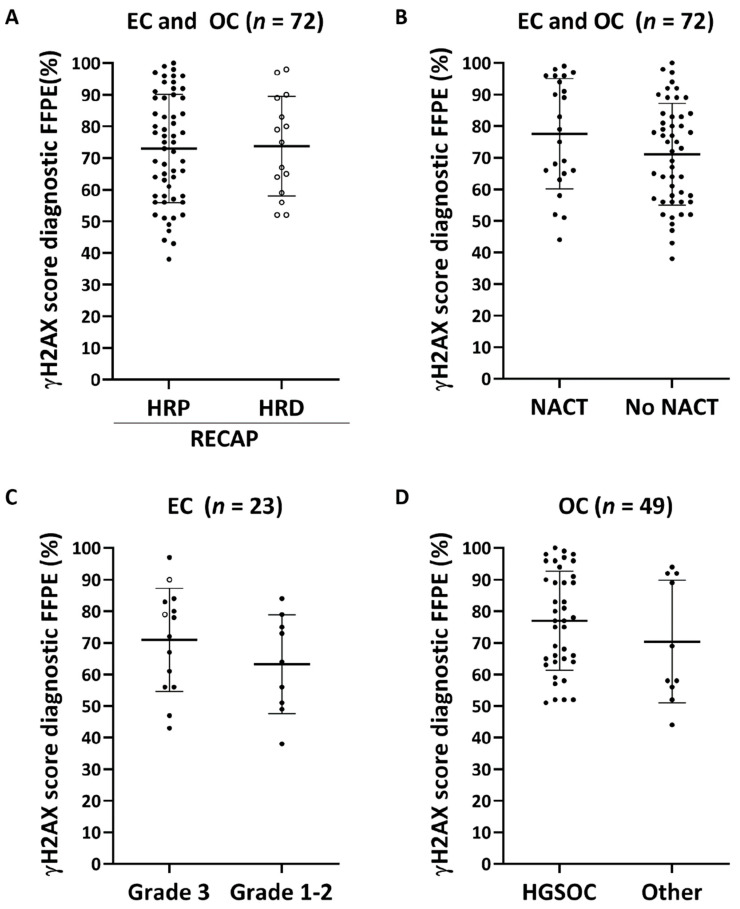
**Assessment of endogenous DNA damage levels in diagnostic FFPE specimens.** The γH2AX score was determined as the percentage of GMN^+^ cells with ≥2 γH2AX foci per nucleus. Means and standard deviations are plotted as horizontal and vertical lines within every scatterplot. Open circles indicate *BRCA* deficient cases (cases with pathogenic variants in *BRCA1/2* or *BRCA1* promotor hypermethylation). HRI cases as determined by the RECAP test were considered HRP in these plots. (**A**) No difference was observed between γH2AX scores of RECAP-HRP versus RECAP-HRD (EC and OC; *p* = 0.708). No significant difference was observed between γH2AX scores of EC and OC diagnostic FFPE specimens due to NACT treatment (*p* = 0.085) (**B**), between grade 3 and grade 1-2 EC (*p* = 0.274) (**C**), or histological subtype OC (*p* = 0.339) (**D**). Other histological subtypes were low-grade serous, endometrioid, mucinous and clear cell OC. Abbreviations: EC = endometrial carcinoma; OC = ovarian carcinoma; RECAP = REcombination CAPacity; HRP = homologous recombination proficient; HRI = homologous recombination intermediate; HRD = homologous recombination proficient; NACT = neoadjuvant chemotherapy.

For all cases that were previously analyzed by the RECAP test, a diagnostic FFPE specimen with more than 70% tumor tissue could be selected (QC1) (Figure 1B). In contrast to the RECAP test, the RAD51-FFPE test requires sufficient levels of endogenous DNA damage in tumor cells to allow evaluation of HR function by analyzing RAD51 protein accumulation at sites of DNA damage (Figure 1A) [41]. To detect endogenous DNA damage in tumors cells, we developed a co-IHC staining protocol for γH2AX/GMN (Figure S1). The γH2AX score, i.e., the percentage of GMN^+^ cells with more than two γH2AX foci/nucleus, was determined for each case. When applying a 25% γH2AX threshold, as described previously [42,43,44], 72/74 (97%) passed QC2 (Figure 1B and Figure 2). We investigated whether differences could be observed between γH2AX scores of diagnostic FFPE specimens when cases were classified as either HR-Proficient (HRP) or HR-Deficient (HRD) based on the results of the RECAP test (RECAP-HRP and RECAP-HRD; Figure 2A and Figure S3). The average γH2AX score of all RECAP-HRP EC and OC specimens (73% (range 38–100%)) was comparable to the average γH2AX score of all RECAP-HRD EC and OC specimens (74% (range 52–98%, *p* = 0.884) (Figure 2A). No differences in γH2AX scores were observed between EC RECAP-HRP and RECAP-HRD cases (*p* = 0.322) (Figure S3A), nor in OC RECAP-HRP and RECAP-HRD cases (*p* = 0.638) (Figure S3B). Next, we explored whether NACT treatment or tumor grade could affect the γH2AX score. EC and OC tumor specimens obtained from patients who were treated with NACT did not yield higher γH2AX scores, (*p* = 0.123) (Figure 2B). In addition, γH2AX scores were not related to tumor grade or the presence of a *TP53* mutation in EC (*p* = 0.274 and *p* = 0.549, respectively) (Figure 2C) nor to histologic subtype in OC (*p* = 0.269) (Figure 2D).

Two out of 49 OC specimens (4%) failed to pass the third quality control step (QC3), i.e., a low GMN count (<40), and were excluded from analysis (Figure 1B). Failure to pass QC3 was not related to NACT treatment or FFPE block age (Figure S4). In total, 70 out of 74 (95%) diagnostic FFPE tumor specimens passed the QC and were successfully analyzed with the RAD51-FFPE test (Figure 1B).

### 3.2. Optimizing RAD51/GMN co-IF for FFPE Tumor Specimens

**Figure 3:**
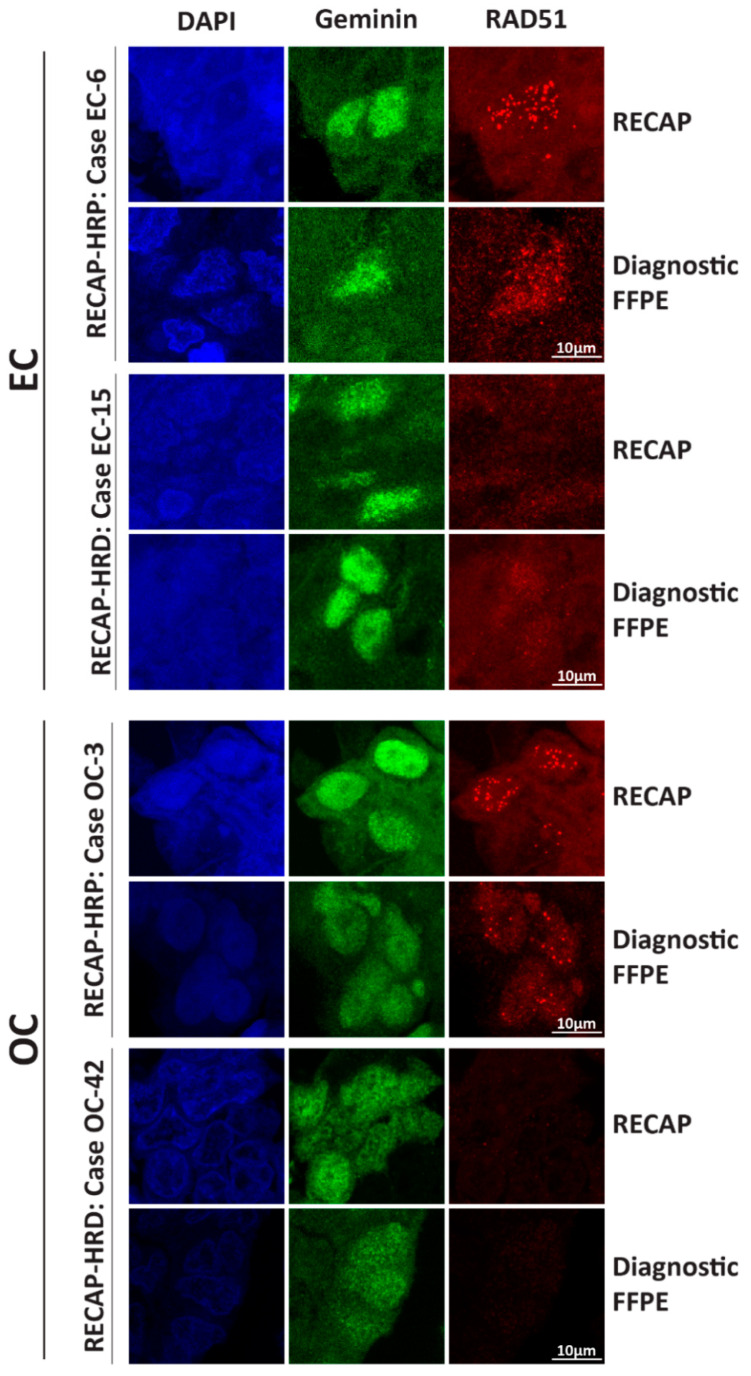
**Microscopy illustration of RECAP versus RAD51-FFPE IF stained slides of EC and OC.** Tumor specimens were stained for geminin, a marker for the G2/S phase, and RAD51 (Diagnostic FFPE: geminin-Novocastra and RAD51-Abcam; RECAP: geminin-ProteinTech and RAD51-GeneTex) (Table S1). Case numbers correspond with case numbers in de Jonge *et al.* and van Wijk *et al.* [31,41]. Abbreviations: EC = endometrial carcinoma; OC = ovarian carcinoma; RECAP = REcombination CAPacity; FFPE = formalin-fixed paraffin-embedded; HRP = homologous recombination proficient; HRD = homologous recombination deficient.

We evaluated the performance of our published RECAP co-IF staining protocol [31,41] for RAD51/GMN (with an anti-RAD51 antibody from GeneTex and an anti-GMN antibody from ProteinTech) in diagnostic FFPE tumor specimens. Using this protocol, we were not able to visualize RAD51 foci in diagnostic FFPE tumor specimens that matched RECAP specimens with abundant numbers of RAD51 foci (RECAP-HRP cases) (Figure S5) [31,41], although sufficiently high endogenous DNA damage levels were present (Figure 2A). Therefore, we tested a variety of alternative primary GMN and RAD51 antibodies (Table S1). GMN antibodies from ProteinTech and Novocastra showed the expected granular nuclear staining. RAD51 foci could solely be visualized with the RAD51 antibody from Abcam (Figure S5). For the subsequent co-IF of diagnostic FFPE specimens the GMN antibody from Novocastra in combination with the RAD51 antibody from Abcam was chosen in order to avoid cross-host interference. Illustrations of staining patterns of diagnostic FFPE EC and OC specimens in the presence and absence of RAD51 foci in GMN^+^ cells are shown in Figure 3. Intra- and inter-tumor variation in the quality and quantity of RAD51 foci was observed among EC and OC specimens, as was aspecific RAD51 staining in some cases (Figure 3 and Figure S6).

The total test duration for the RAD51-FFPE test is five days, in contrast to ten days for the RECAP test (Table S2). In addition, the RAD51-FFPE test can be performed at lower cost (Table S3).

### 3.3. RAD51-FFPE Test Parameters: Calibration of the RAD51 Foci Cut-Off and HRD Threshold

Since numbers of RAD51 foci varied considerably in GMN^+^ cells, we calculated the sensitivity and specificity of the RAD51-FFPE test using different RAD51 foci cut-offs and HRD thresholds. For each GMN^+^ cell, we scored the number of RAD51 foci per nucleus (0, 1, 2, 3, 4, ≥5 foci). We next determined the percentage of GMN^+^ cells that were RAD51^+^ based on different RAD51 foci number thresholds. In addition, application of four HRD thresholds was evaluated, ranging from 5% till 20% (Table 1). To determine which combination of parameters (i.e., RAD51 foci cut-off and HRD threshold) yielded the highest sensitivity and specificity of the RAD51-FFPE test, results of *BRCA* deficient (pathogenic variants in *BRCA1/2* or *BRCA1* promotor hypermethylation) and RECAP-HRD cases were used as a reference. When considering *BRCA* deficient cases as HRD, the optimal sensitivity and specificity was reached with a RAD51 foci cut-off of ≥2 foci and an HRD threshold of 15% (sensitivity = 90%, specificity = 68%). The same combination of parameters resulted in the highest sensitivity and specificity (sensitivity = 87%, specificity = 73%) when RECAP-HRD (including all *BRCA* deficient) cases were considered to represent true HRD cases (Table 1). By applying test parameters of ≥2 foci and an HRD threshold of 15% the overall agreement in HR-class assignment between the RECAP test and RAD51-FFPE test was 83% for EC cases, 72% for OC cases and 76% for the total study cohort (EC and OC combined) (Table S4).

**Figure 4:**
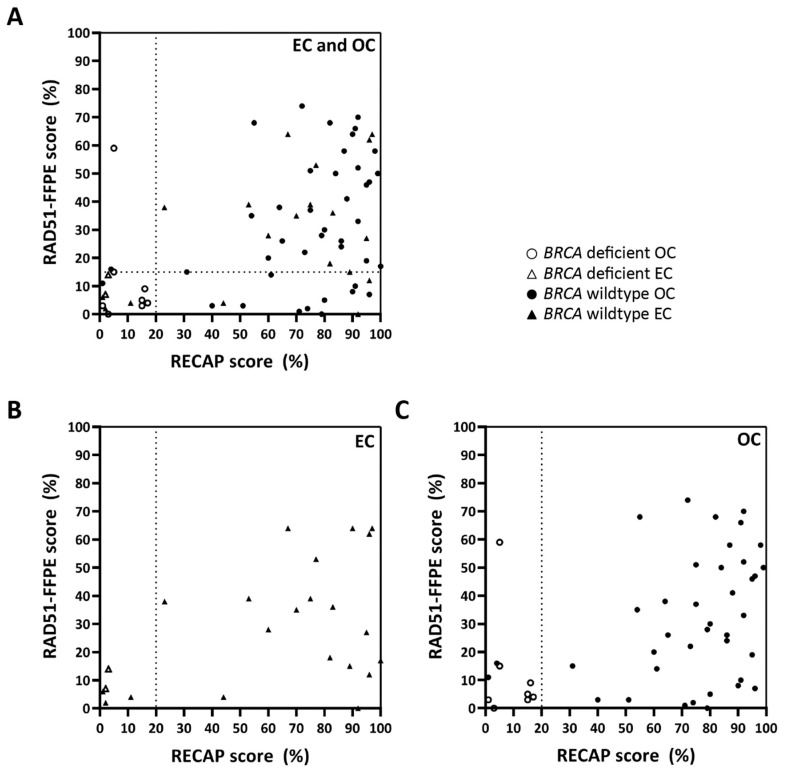
**Correlation RAD51-FFPE scores with RECAP scores in EC and OC.** RECAP scores were calculated as the percentage of GMN^+^ cells with ≥5 RAD51 foci. RAD51-FFPE scores were calculated as the percentage of GMN^+^ cells with ≥2 RAD51 foci. The 20% HRD threshold for HR classification of RECAP specimens is indicated with a dotted vertical line. RAD51-FFPE scores significantly correlated with RECAP scores for EC and OC combined (**A**) (*n* = 70; Pearson R^2^ = 0.20, *p* = 0.0001), for EC (**B**) (*n* = 23; Pearson R^2^ = 0.22, *p* = 0.025) and for OC (**C**) (*n* = 47; Pearson R^2^ = 0.19, *p* = 0.0023). One OC case (OC-45) with a pathogenic variant in *BRCA1* and classified as HRD by the RECAP test (RECAP score 5%) was a clear outlier in our study, as it had a RAD51-FFPE score of 59%. Details for this case can be found in Table S5. Abbreviations: EC = endometrial carcinoma; OC = ovarian carcinoma; RECAP = REcombination CAPacity; FFPE = formalin-fixed paraffin-embedded; GMN = geminin; *BRCA* deficient = cases with pathogenic variants in *BRCA1/2* or *BRCA1* promotor hypermethylation.

In an exploratory manner, we tested whether absolute RAD51-FFPE scores correlated with absolute RECAP scores, without the application of an HRD threshold. When a RAD51 foci cut-off of ≥2 foci was applied, a significant correlation was identified between RAD51-FFPE scores and matching RECAP scores [31,41] for both EC and OC (EC and OC combined: Pearson R^2^ = 0.20, *p* = 0.0001; EC: Pearson R^2^ = 0.22, *p* = 0.0245; OC: Pearson R^2^ = 0.19, *p* = 0.0023) (Figure 4A–C).

## 4. Discussion

The RAD51/GMN co-IF on FFPE specimens has previously been described for breast tumor specimens [42,43], but optimal test parameters (γH2AX threshold, RAD51 foci cut-off and HRD threshold) were not extensively defined and not available for EC and OC. Here, we show that, using an optimized and calibrated protocol, diagnostic FFPE tumor specimens of EC and OC can be successfully analyzed with the RAD51-FFPE test. In addition, we performed a side-by-side comparison of FFPE tumor specimens with matched specimens for which HR status was determined using a RAD51-based test that required *ex vivo* irradiation of fresh tissue (RECAP test). We show that the RAD51-FFPE test detected *BRCA* deficient tumors and RECAP-HRD tumors with high sensitivity and can be applied to the majority of diagnostic FFPE specimens from EC and OC.

For optimal performance of the RAD51-FFPE test, we applied two additional quality control steps compared to the RECAP test. Firstly, a tumor block with >70% vital appearing tumor tissue was selected by a gynecopathologist (TB), based on a H&E stained slide. Secondly, we determined whether sufficient levels of endogenous DNA damage were present, by implementing a γH2AX score (percentage of γH2AX^+^/GMN^+^ cells based on ≥2 foci per nucleus). In our EC and OC cohorts almost all (97%) specimens had a γH2AX score above 25%, similar to what has been observed for breast FFPE specimens [42,43,44]. Interestingly, these findings suggest that sufficient endogenous DNA damage for an adequate RAD51-FFPE test is present in the vast majority of diagnostic FFPE tumor blocks. Consequently, in the future it may be acceptable to use γH2AX staining only to confirm sufficient levels of endogenous DNA damage in cases which are classified as HRD.

In this study, we had the unique opportunity to compare RAD51-FFPE results with RECAP test results. We observed that the number of RAD51 foci in the RAD51-FFPE test is usually lower and that the foci are generally smaller as compared to the RECAP test. We therefore recalibrated test parameters (RAD51 foci cut-off and HRD threshold) to reach optimal sensitivity and specificity. A RAD51 foci cut-off of ≥2 with an HRD threshold of 15% resulted in the highest sensitivity for the identification of *BRCA* deficient (90%) and RECAP-HRD (87%) cases, and a specificity of 68% and 73%, to identify wt*BRCA* and RECAP-HRP cases, respectively. The relatively low specificity was due to a substantial fraction of RECAP-HRP cases that displayed low RAD51-FFPE scores. This discrepancy in HRD score appears not to be caused by the absence of endogenous DNA damage as γH2AX levels in these samples were similar to samples with high RAD51-FFPE scores. It is, however, unclear whether the DNA damage detected by γH2AX staining in the RAD51-FFPE low samples represents valid substrates for HR.

The RAD51-FFPE test still requires additional improvements before it can be advanced to routine diagnostics with automated scanning and scoring as most prominent avenue for further exploration. However, multiple observers will probably remain required for manual analysis of cases with suboptimal staining (autofluorescence, aspecific RAD51 staining and/or strong pan-nuclear RAD51 staining). Alternatively, development of a high quality co-IHC protocol for RAD51/GMN may overcome above listed limitations related to the use of fluorescent antibodies.

This study provides a solid framework for advancing the RAD51-FFPE test into a diagnostic test as it is a sensitive, rapid and low-cost test. As it can be performed on small tissue specimens, including biopsies, it can be used both in first line as well as recurrent setting. This is particularly important, since PARPi have now been approved in both settings [2,3]. Although the use of platinum sensitivity as a biomarker for PARPi response has proven its value in the recurrent setting, platinum sensitivity in the first line setting might be less informative considering that many patients will undergo complete debulking and the direct effect of platinum treatment on tumor response is more difficult to assess.

Given the high sensitivity of the test, it is particularly suited in the first line setting to facilitate the identification of patients who might benefit from PARPi and/or platinum-based chemotherapy and as prescreening tool to select tumors which are most likely to have pathogenic variants in HR-related genes. In the recurrent setting, where DNA-based HRD tests might be less informative due to their inability to identify tumors with reversal of the HRD phenotype, the RAD51-FFPE test performed on biopsies might be the preferred test to guide therapy choice.

The performance of the current RAD51-FFPE test parameters requires, and is currently undergoing, validation in independent large randomized study cohorts, for which both DNA-based HRD scores (e.g., the MyChoice HRD or HRDetect) and the response to therapy are available. It is conceivable that further refinement of the RAD51-FFPE test characteristics will be required before optimal treatment benefit prediction is achieved, a process that is currently also ongoing for the calibration of HRD thresholds in DNA-based HRD tests [45,46].

## 5. Conclusions

In this study, an improved protocol for the RAD51-FFPE test was established and test parameters were calibrated using the RECAP test to identify functional HRD in EC and OC. The threshold settings for HR status assignment can serve as basis for advancing the RAD51-FFPE test from a research tool towards a clinically applicable diagnostic test.

## Figures and Tables

**Table 1:** **Concordance in HRD classification between RECAP and RAD51-FFPE test results when applying various HRD thresholds and RAD51 foci number cut-offs.** The HR group classification of the RECAP test was based on a 20% HRD threshold with a RAD51 foci cut-off of ≥ 5 (HRD ≤ 20%; HRP ≥ 20%). RECAP-HRI cases were considered as HRP in this analysis. The test parameters with the highest sensitivity and specificity for all cases are highlighted in bold. Sub analysis of EC and OC cases is represented in Table S4.

RAD51-FFPE Test Parameters		Sensitivity/Specificity EC and OC Combined (%)			
HRD Classification Threshold	FFPE RAD51 Foci Number	Sensitivity *BRCA* Deficient *n* = 10	Sensitivity RECAP-HRD *n* = 15	Specificity wt*BRCA* *n* = 60	Specificity RECAP-HRP *n* = 55
≤5%	1	20	20	87	89
	2	50	47	83	87
	3	60	60	77	82
	4	60	60	67	71
	≥5	60	60	63	67
≤10%	1	60	60	82	85
	2	70	67	78	80
	3	70	73	70	75
	4	90	87	57	60
	≥5	90	93	53	58
**≤15%**	1	60	67	77	82
	**2**	**90**	**87**	**68**	**73**
	3	90	93	58	64
	4	90	93	42	45
	≥5	90	93	38	42
≤20%	1	80	80	72	76
	2	90	93	60	65
	3	90	93	47	51
	4	90	93	37	40
	≥5	90	93	30	33

Abbreviations: EC = endometrial carcinoma; OC = ovarian carcinoma; RECAP = REcombination CAPacity; FFPE = formalin-fixed paraffin-embedded; HR = homologous recombination; HRD = homologous recombination deficient; HRI = homologous recombination intermediate; *BRCA* deficient = cases with pathogenic variants in *BRCA1/2* or *BRCA1* promotor hypermethylation.

## Data Availability

Data is contained within the article or supplementary material. The RECAP data presented in this study are available in de Jonge *et al*. and van Wijk *et al*. [31,41].

## Supplementary Materials

The following are available online at https://www.mdpi.com/article/10.3390/cancers13122994/s1, Figure S1: H&E and γH2AX/Geminin co-IHC stained diagnostic FFPE specimens, Figure S2: Interobserver variability of RAD51-FFPE scores for EC and OC cases, Figure S3: Sufficient endogenous DNA damage as represented by high γH2AX scores was observed in both EC and OC diagnostic FFPE specimens, Figure S4: FFPE block age of diagnostic FFPE EC and OC specimens, Figure S5: Primary antibody selection for geminin and RAD51 co-immunofluorescence (co-IF) staining on diagnostic FFPE specimens, Figure S6: Microscopy images of RECAP versus RAD51-FFPE immunofluorescence (IF) stained slides of EC and OC showing intra- and inter-tumor heterogeneity, Table S1: Primary antibodies tested for geminin and RAD51 co-IF Table S2: Workload RECAP test versus RAD51-FFPE test, Table S3: Costs RECAP test versus RAD51-FFPE test, Table S4: HR class classification agreement between the RECAP test and RAD51-FFPE test in EC, OC and the total study cohort, Table S5: Cases with a RAD51-FFPE score differences of >30% between observer 1 and observer 2 for which a third observer was consulted.
