# Supplementary material for: The RAD51-FFPE Test; Calibration of a Functional Homologous Recombination Deficiency Test on Diagnostic Endometrial and Ovarian Tumor Blocks
Source: Cancers (Basel). 2021 Jun 15;13(12):2994. doi: 10.3390/cancers13122994 (PMC8232577; doi:10.3390/cancers13122994)
Supplement: Supplementary file 1 [file cancers-13-02994-s001.zip › cancers-1229686-supplementary.pdf]

cancers

Supplementary Files

The RAD51-FFPE Test; Calibration of a Functional Homologous Recombination Deficiency Test on Diagnostic Endometrial and Ovarian Tumor Blocks

Lise M. van Wijk, Claire J.H. Kramer, Sylvia Vermeulen, Natalja T. ter Haar, Marthe M. de Jonge, Judith R. Kroep, Cor D. de Kroon, Katja N. Gaarenstroom, , Harry Vrieling, Tjalling Bosse and Maaike P.G. Vreeswijk

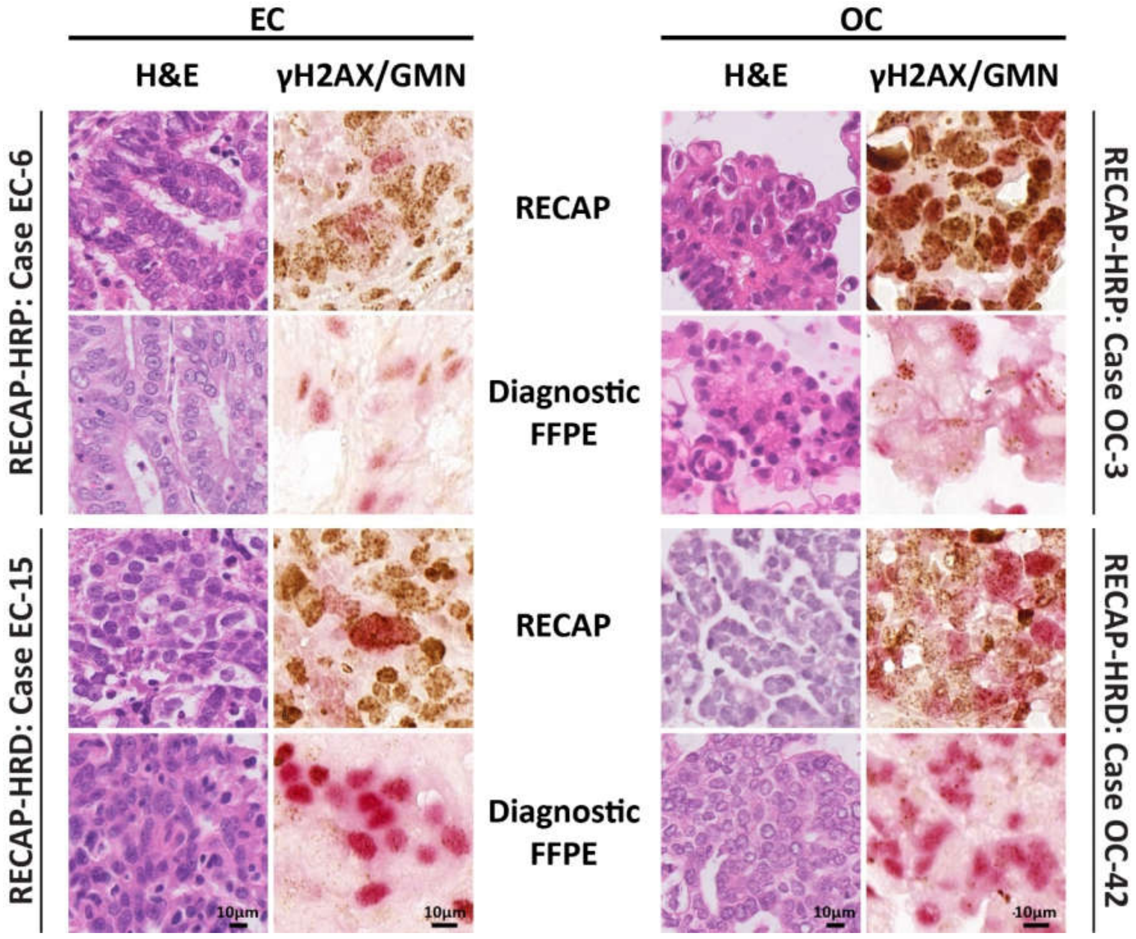

Figure S1. **H&E and γH2AX/Geminin co-IHC stained diagnostic FFPE specimens.** Case numbers correspond with case numbers in de Jonge et al. and van Wijk et al. [25,35]. HRP and HRD classification is based on RECAP test results. Images are scans from γH2AX/GMN co-IHC slides (γH2AX in brown and GMN in pink). Abbreviations: EC = endometrial carcinoma; OC = ovarian carcinoma; GMN = geminin; H&E = hematoxylin and eosin; RECAP = REcombination CAPacity; FFPE = formalin-fixed paraffin-embedded; HRP = homologous recombination proficient; HRD = homologous recombination deficient..

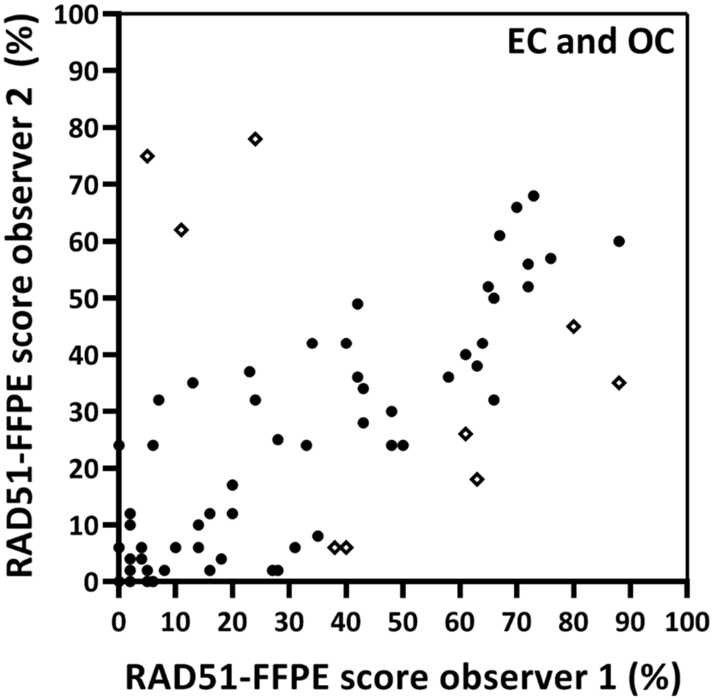

Figure S2. Interobserver variability of RAD51-FFPE scores for EC and OC cases. For nine OC cases (indicated as open diamonds) a RAD51-FFPE score difference of > 30% between two observers was observed, resulting in a moderate agreement for HR class assignment of RAD51-FFPE cases when an HRD threshold of 15% was applied (foci cut-off ≥ 2) (κ = 0.50). The RAD51-FFPE scores of observer 1 and observer 2 were significantly correlated ($p < 0.0001$). Final RAD51-FFPE scores for discrepant cases were obtained after evaluation of the RAD51-FFPE score from a third independent observer (Table S5). The median of final RAD51-FFPE scores (EC and OC combined) differed by 9% (range: 0–28%) between two independent observers. Abbreviations: EC = endometrial carcinoma; OC = ovarian carcinoma; FFPE = formalin-fixed paraffin-embedded.

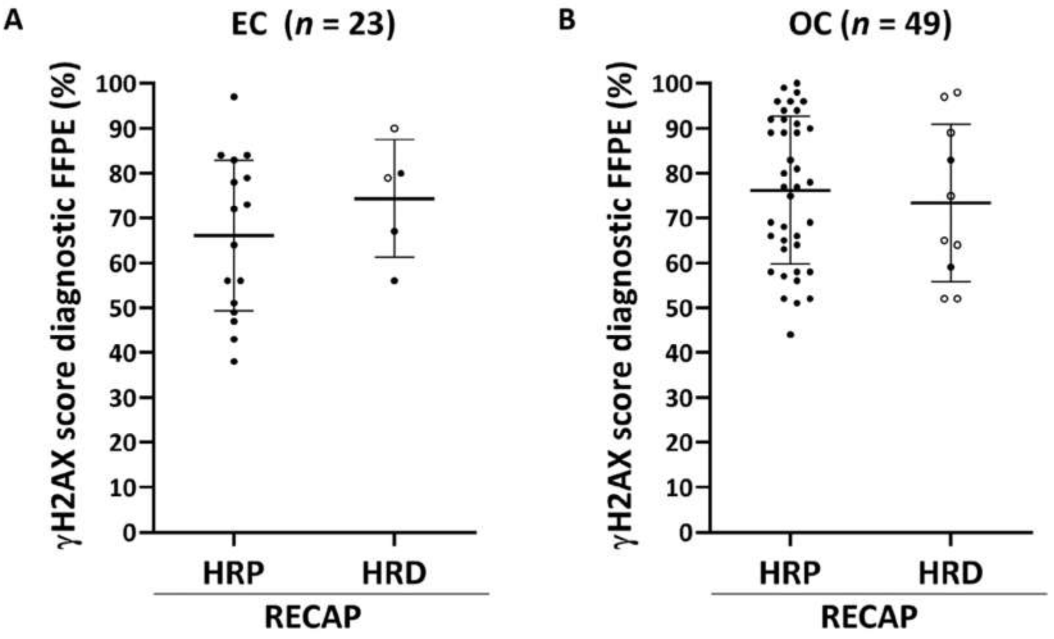

Figure S3. Sufficient endogenous DNA damage as represented by high γH2AX scores was observed in both EC and OC diagnostic FFPE specimens. The γH2AX score was determined as the percentage of GMN^+ cells with ≥ 2 γH2AX foci per nucleus. Means and standard deviations are plotted as horizontal and vertical lines, respectively. Open circles indicate BRCA-deficient cases (pathogenic variants in BRCA1/2 or BRCA1 promotor hypermethylation). No significant difference between γH2AX scores was observed between RECAP-HRP and RECAP-HRD cases for EC (**A**) (p = 0.322) and OC (**B**) (p = 0.638). Abbreviations: EC = endometrial carcinoma; OC = ovarian carcinoma; RECAP = REcombination CAPacity; HRP = homologous recombination proficient; HRD = homologous recombination deficient.

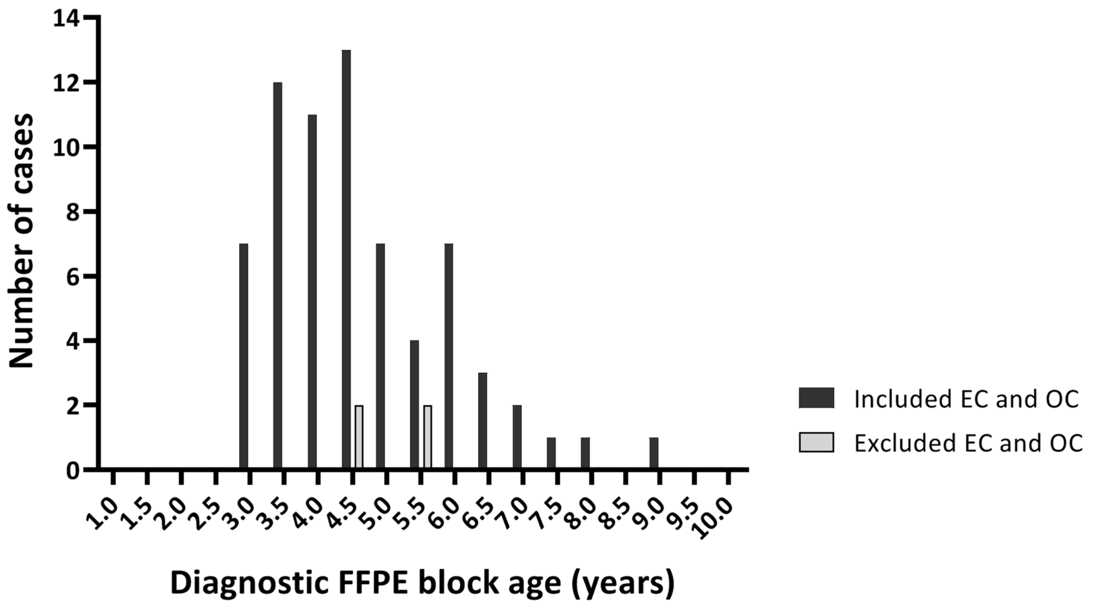

Figure S4. FFPE block age of diagnostic FFPE EC and OC specimens. No difference in block age between included and excluded cases was observed (EC and OC combined) (Mann-Whitney Rank Sum Test $p = 0.328$). Abbreviations: EC = endometrial carcinoma; OC = ovarian carcinoma; FFPE = formalin-fixed paraffin-embedded.

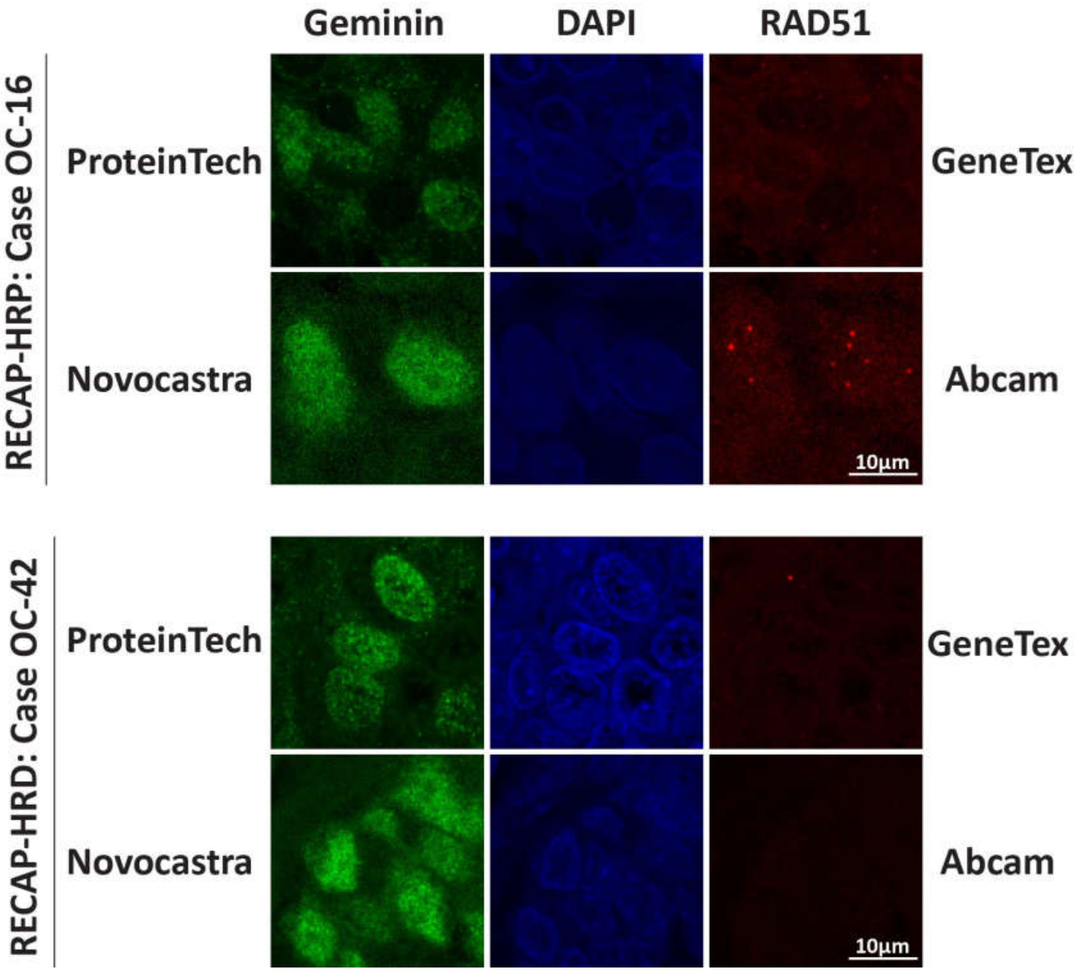

Figure S5. Primary antibody selection for geminin and RAD51 co-immunofluorescence (co-IF) staining on diagnostic FFPE specimens. Case numbers correspond with case numbers in de Jonge et al. and van Wijk et al. [25,35]. Details about the antibodies from ProteinTech, Novocastra, GeneTex and Abcam are provided in Materials and Methods sections 2.2 and 2.4 and in Table S1. Abbreviations: OC = ovarian carcinoma; RECAP = REcombination CAPacity; FFPE = formalin-fixed paraffin-embedded; HRP = homologous recombination proficient; HRD = homologous recombination deficient.

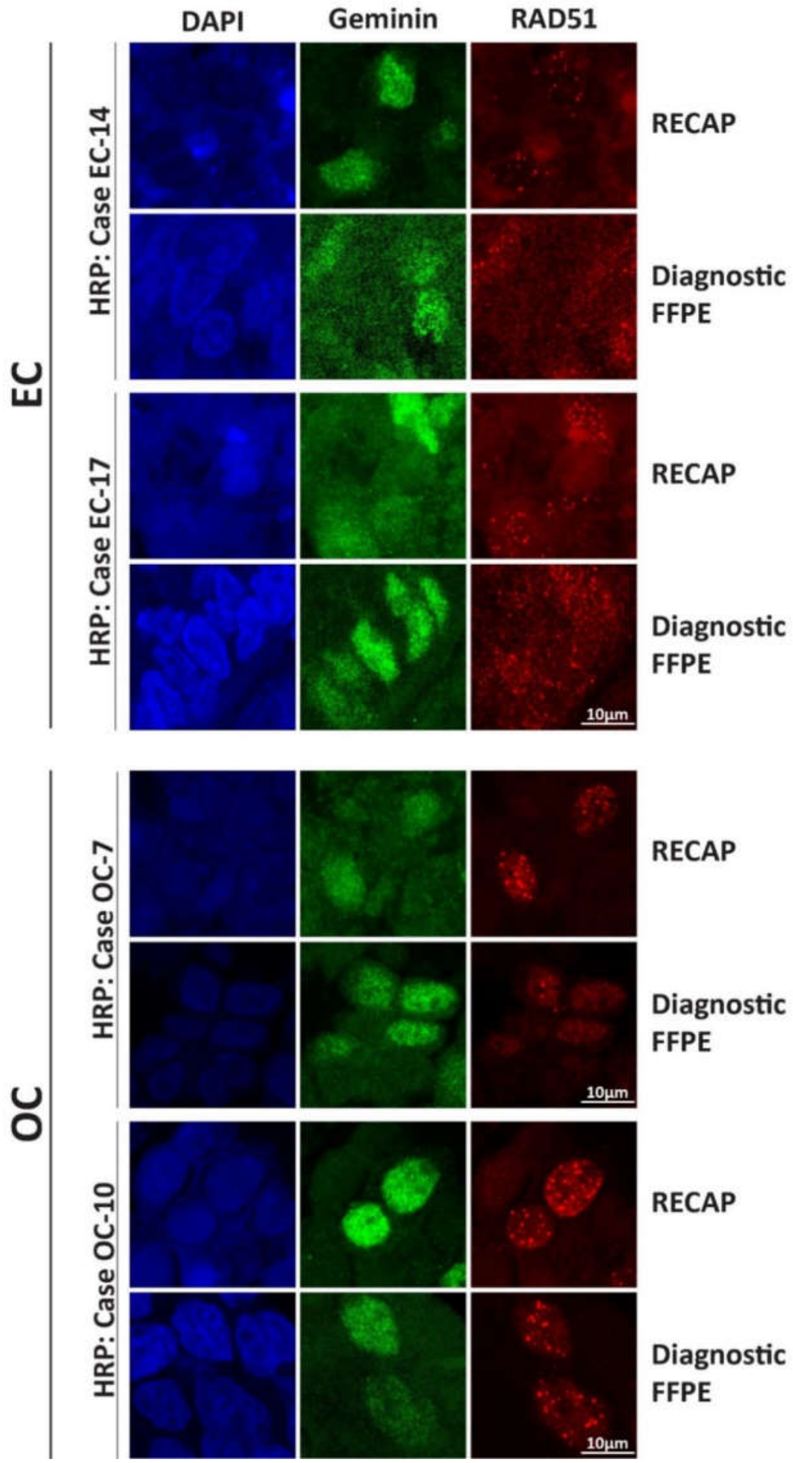

Figure S6. Microscopy images of RECAP versus RAD51-FFPE immunofluorescence (IF) stained slides of EC and OC showing intra- and inter-tumor heterogeneity. Case numbers correspond with case numbers in de Jonge et al. and van Wijk et al. [25,35]. RECAP slides were stained according to the co-IF protocol as published in de Jonge et al [25]. RAD51-FFPE slides were stained according to the co-IF protocol as described in section 2.4 of the Materials and Methods. Abbreviations: EC = endometrial carcinoma; OC = ovarian carcinoma; RECAP = REcombination CAPacity; FFPE = formalin-fixed paraffin-embedded; HRP = homologous recombination proficient.

Table S1. Primary antibodies tested for geminin and RAD51 co-IF. Dilutions as shown in the table were the most optimal dilutions as determined by antibody titration. Antibodies selected for the RAD51-FFPE test are indicated in bold. Abbreviations: co-IF = co-immunofluorescence.

COPYRIGHT: © 2021 BY THE AUTHORS. LICENSEE MDPI, BASEL, SWITZERLAND. THIS ARTICLE IS AN OPEN ACCESS ARTICLE DISTRIBUTED UNDER THE TERMS AND CONDITIONS OF THE CREATIVE COMMONS ATTRIBUTION (CC BY) LICENSE (HTTP://CREATIVECOMMONS.ORG/LICENSES/BY/4.0/).

ANTIBODY	PROVIDER	REFERENCE	SPECIES	CLONALITY	DILUTION USED FOR IF	OBSERVATION
**ANTI-GEMININ**	**Novocastra**	**NCL-L**	**Mouse**	**Monoclonal**	**1 in 60**	**Granular staining of the nucleus**
	Proteintech*	10802-1-AP	Rabbit	Polyclonal	1 in 400	Granular staining of the nucleus
	LSbio	LS-B6226	Mouse	Monoclonal	1 in 100	Aspecific staining
	Abcam	ab104306	Mouse	Monoclonal	1 in 100	No GMN staining
**ANTI-RAD51**	**Abcam**	**ab133534**	**Rabbit**	**Monoclonal**	**1 in 1000**	**Foci and occasionally pan-nuclear/aspecific staining**
	GeneTex*	GTX70230	Mouse	Monoclonal	1 in 400	No foci
	Bio Academia	70-001	Rabbit	Polyclonal	1 in 1000	No foci and a lot of aspecific staining

Abbreviations: co-IF = co-immunofluorescence, Antibodies for the REcombination CAPacity (RECAP) test.

Table S2. Workload RECAP test versus RAD51-FFPE test. The workload for both the RECAP test and RAD51-FFPE test are estimations based on working procedures in the Leiden University Medical Center (LUMC), Leiden, the Netherlands.

	RECAP TEST						RAD51-FFPE TEST				
	**Activity**	**Hands-on time per sample**	**Hands-on time per full run (25 samples)**	**Complete time per sample**	**Complete time per full run (25 samples)**		**Activity**	**Hands-on time per sample**	**Hands-on time per full run (25 samples)**	**Complete time per sample**	**Complete time per full run (25 samples)**
**GENERAL**	Tissue embedding	10 min	45 min	10 min	45 min	**GENERAL**					
	Paraffin block slicing	10 min	60 min	10 min	60 min		Paraffin block slicing	10 min	60 min	10 min	60 min
**QUALITY CONTROL**	H&E staining (machine)	10 min	10 min	10 min	10 min	**QUALITY CONTROL**	H&E staining (machine)	10 min	10 min	10 min	10 min
	γH2AX IHC staining	150 min	150 min	1338 min	1338 min		γH2AX/ GMN co-IHC staining	150 min	150 min	1338 min	1338 min
	p53 IHC staining	150 min	150 min	1338 min	1338 min						
	Pathologist H&E, γH2AX and P53 review (5 min per slide)	15 min	135 min	15 min	135 min		Pathologist H&E and γH2AX/ GMN review (5 min per slide)	10 min	250 min	10 min	250 min
**RECAP TEST**	Slicing tumor tissue	10 min	250 min	10 min	250 min	**RAD51-FFPE TEST**					
	Preparation culture system	15 min	30 min	15 min	30 min						
	Cryopreser-vation tissue	20 min	60 min	20 min	60 min						
	Irradiation	2 min	18 min	10 min	90 min						
	Tissue fixation	10 min	60 min	10 min	60 min						
	RAD51/GMN co-IF staining	150 min	150 min	378 min	378 min		RAD51/ GMN co-IF staining	120 min	120 min	211 min	211 min
	Co-IF slide scoring	15 min	375 min	15 min	375 min		Co-IF slide scoring	20 min	500 min	30 min	750 min
	**10 days for full run**	**9h 27 min**	**24h 53 min**	**53h**	**69h 29 min**		**5 days for full run**	**5h 20 min**	**18h 10 min**	**26h 50 min**	**43h 39 min**

Abbreviations: RECAP = REcombination CAPacity; FFPE = formalin-fixed paraffin-embedded; co-IF = co-immunofluorescence; co-IHC = co-immunohistochemistry; min = minutes; GMN = geminin; h = hours; H&E = hema-toxylin and eosin; o/n = overnight.

Table S3. Costs RECAP test versus RAD51-FFPE test. The total cost prices (excl. personnel costs) for both the RECAP test and RAD51-FFPE test are indications based on European market prices in December 2020.

	RECAP TEST					RAD51-FFPE TEST			
	**Product**	**Costs per sample**	**Costs per full run (25 samples)**	**Costs per sample in full run**		**Product**	**Costs per sample**	**Costs per full run (25 samples)**	**Costs per sample in full run**
**GENERAL**	Paraffin block (embedding, cassette, foams)	€ 3,00	€ 75,00	€ 3,00	**GENERAL**				
	Formalin	€ 0,08	€ 0,08	€ 0,00		Formalin	€ 0,08	€ 0,08	€ 0,00
	Disposables (pipet tips, wells-plate, falcon tubes)	€ 0,20	€ 5,00	€ 0,20		Disposables (pipet tips, wells-plate, falcon tubes)	€ 0,20	€ 5,00	€ 0,20
	Glassware (microscope slides, coverslips)	€ 0,30	€ 7,50	€ 0,30		Glassware (microscope slides, coverslips)	€ 0,30	€ 7,50	€ 0,30
**QUALITY CONTROL**	H&E machinal costs	€ 0,11	€ 2,75	€ 0,11	**QUALITY CONTROL**	H&E machinal costs	€ 0,11	€ 2,75	€ 0,11
	Primary antibodies (γH2AX and p53)	€ 0,05	€ 1,25	€ 0,05		Primary antibodies (γH2AX and Geminin)	€ 0,07	€ 1,82	€ 0,07
	Secondary antibodies (DAB+ substrate chromogen system)	€ 1,00	€ 25,00	€ 1,00		Secondary antibodies (DAB+ substrate chromogen system)	€ 1,00	€ 25,00	€ 1,00
	Mounting medium (Surgipath Micromount)	€ 0,50	€ 12,50	€ 0,50		Mounting medium (Surgipath Micromount)	€ 0,50	€ 12,50	€ 0,50
**RECAP TEST PROCEDURE**	OSE medium	€ 0,25	€ 6,25	€ 0,25	**RAD51-FFPE TEST PROCEDURE**				
	DNAse	€ 0,76	€ 18,88	€ 0,76		DAKO Antibody Diluent	€ 4,17	€ 4,17	€ 0,17
	Antigen retrieval buffer (Tris/EDTA)	€ 4,92	€ 4,92	€ 0,20		DAKO Antigen Retrieval Buffer	€ 21,56	€ 21,56	€ 0,86
	Wash buffer (PBS)	€ 2,50	€ 2,50	€ 0,10		DAKO Wash Buffer	€ 8,80	€ 8,80	€ 0,35
	Primary antibodies (RAD51 and geminin)	€ 1,80	€ 45,00	€ 1,80		Primary antibodies (RAD51 and geminin)	€ 2,34	€ 58,61	€ 2,34
	Secondary antibodies (Alexa Fluor 488 and Alexa Fluor 555)	€ 0,10	€ 2,50	€ 0,10		Secondary antibodies (Alexa Fluor 488 and Alexa Fluor 555)	€ 0,10	€ 2,50	€ 0,10
	ProLong Gold Antifade mount with DAPI	€ 1,30	€ 32,50	€ 1,30		ProLong Gold Antifade mount with DAPI	€ 1,30	€ 32,50	€ 1,30
	**Total cost price**	€ 16,87	€ 241,63	**€ 9,67**		**Total cost price**	€ 40,53	€ 182,79	**€ 7,30**

Abbreviations: NGS = next-generation sequencing; H&E = hematoxylin and eosin; RECAP = REcombination CAPacity.

.

Table S4. HR class classification agreement between the RECAP test and RAD51-FFPE test in EC, OC and the total study cohort. The test parameters with the highest sensitivity and specificity for all cases are highlighted in light grey.

RAD51-FFPE	test parameters	HR class assignment agreement between RECAP test and RAD51-FFPE test (%)		
**HRD threshold**	**FFPE RAD51 foci number**	**EC** *n* = 23	**OC** *n* = 47	**EC and OC combined** *n* = 70
≤ 5%	**1**	74	72	73
	**2**	78	77	77
	**3**	83	72	76
	**4**	70	66	67
	**≥5**	65	64	64
≤ 10%	**1**	87	77	80
	**2**	87	72	77
	**3**	78	72	74
	**4**	70	64	66
	**≥5**	70	64	66
≤ 15%	**1**	87	74	79
	**2**	83	72	76
	**3**	74	68	70
	**4**	61	53	56
	**≥5**	57	51	53
≤ 20%	**1**	87	72	77
	**2**	74	70	71
	**3**	70	55	60
	**4**	57	49	51
	**≥5**	48	45	46

Abbreviations: EC = endometrial carcinoma; OC = ovarian carcinoma; RECAP = REcombination CAPacity; FFPE = formalin-fixed paraffin-embedded; HRP = homologous recombination proficient; HRD = homologous recombination deficient.

Table S5. Cases with a RAD51-FFPE score differences of > 30% between observer 1 and observer 2 for which a third observer was consulted. Case numbers correspond with case numbers in de Jonge et al. and van Wijk et al. [25,35]. Since we had to adapt our preferred routine (consensus meeting with the two observers at the microscope when a difference of > 30% was observed between the scores of two observers) due to the COVID-19 regulations in our laboratory, we consulted a third observer to score the slide. Final RAD51-FFPE scores were calculated as the average of the two closest RAD51-FFPE scores (Materials and Methods section 2.6). With this strategy one clear outlier was identified (Case OC-45; RECAP-HRD, 5% and RAD51-FFPE HRP, 59%). After unblinding the data this specific outlier was reanalysed and we concluded that the aspecific RAD51 staining in the RAD51-FFPE slide was likely incorrectly scored as RAD51 foci.

RAD51-FFPE score (%)				Observation sub-optimal staining		
**Case**	**Observer 1**	**Observer 2**	**Observer 3**	**Strong auto-fluorescence**	**Aspecific RAD51 staining**	**Strong pan-nuclear RAD51 staining**
OC-1	63	18	37		X	X
OC-4	75	5	8		X	X
OC-7	6	38	28			X
OC-9	40	6	64			X
OC-15	88	35	17		X	
OC-30	26	61	25	X		
OC-35	78	24	58	X		
OC-36	80	45	25	X		
OC-45	62	11	55		X	

Abbreviations: OC = ovarian carcinoma; FFPE = formalin-fixed paraffin-embedded.
